# Selective enrichment of TCF4 in GABAergic neurons during postnatal primate development

**DOI:** 10.3389/fnana.2026.1720211

**Published:** 2026-05-19

**Authors:** Alain C. Burette, Rebecca I. Hipp, Sarah Salvador, Chavely Gonzalez Ramirez, Ridthi K. R. Patel, Naomi R. Salvador, Katherine A. Dasher, Jeff Bennett, David G. Amaral, Benjamin D. Philpot

**Affiliations:** 1 Neuroscience Center, University of North Carolina at Chapel Hill, Chapel Hill, NC, United States; 2 Department of Cell Biology and Physiology, University of North Carolina at Chapel Hill, Chapel Hill, NC, United States; 3 Department of Psychiatry and Behavioral Sciences, MIND Institute, University of California, Davis, Davis, CA, United States; 4 California National Primate Research Center, University of California, Davis, Davis, CA, United States; 5 Carolina Institute for Developmental Disabilities, University of North Carolina at Chapel Hill, Chapel Hill, NC, United States

**Keywords:** GABAergic interneurons, gene expression, macaque, neocortex, neurodevelopment, Pitt-Hopkins syndrome, schizophrenia, TCF4

## Abstract

The transcription factor 4 (*TCF4*) gene is essential for brain development, and its disruption causes Pitt-Hopkins syndrome. Common genetic variants in *TCF4* also confer risk for schizophrenia and related psychiatric disorders. While the developmental roles of TCF4 are well established, its postnatal functions remain poorly defined, particularly during the prolonged maturation of the primate neocortex, when many neuropsychiatric symptoms first emerge. Here, we mapped cell-type-specific TCF4 expression across postnatal development in rhesus macaque neocortex using immunofluorescence, and *in situ* hybridization. We also re-analyzed public bulk-tissue and single-nucleus transcriptomic datasets spanning 16 brain regions in both humans and macaques. TCF4 was predominantly neuronal, with minimal expression in microglia, oligodendrocytes, and astrocytes. In late gestation, TCF4 was broadly expressed in excitatory and inhibitory neurons, but expression declined with maturation and became selectively enriched in inhibitory populations. By young adulthood, nuclear TCF4 levels were highest in somatostatin-positive and vasoactive intestinal peptide-positive interneurons, intermediate in parvalbumin interneurons (~6-fold above non-GABAergic cells), and lowest in cholecystokinin interneurons (~4-fold above non-GABAergic cells). This interneuron-subtype hierarchy was independently reproduced by single-nucleus RNA sequencing across all neocortical regions. Together, these findings position mature GABAergic interneurons as the principal site of TCF4 function in the primate neocortex, providing a cellular framework for linking TCF4 dysfunction to cortical circuit imbalance in Pitt-Hopkins syndrome and psychiatric disease.

## 1 Introduction

Transcription factor 4 (TCF4, OMIM 602272 also known as ITF2, SEF2, E2-2) is a key regulator of neurodevelopment, controlling gene expression essential for neurogenesis (Fischer et al., 2014; Li et al., 2019), neuronal differentiation, and the maturation of neurons and oligodendrocytes (Phan et al., 2020; Wedel et al., 2020). Through these mechanisms, TCF4 shapes brain architecture by influencing cerebral cortical layering (Chen et al., 2016; Li et al., 2019), hippocampal development (Wang et al., 2020), and the formation of major fiber tracts (Mesman et al., 2020). The importance of TCF4 during brain development is evident in specific human diseases associated with mutations or dysregulation of this gene. Pitt-Hopkins syndrome (PTHS OMIM #610954) is the most well-defined condition directly caused by TCF4 dysfunction (Amiel et al., 2007; Brockschmidt et al., 2007; Zweier et al., 2007), arising from heterozygous mutations or deletions at chromosome 18q21.2. These genetic alterations result in *TCF4* haploinsufficiency or production of defective proteins, often affecting the critical bHLH domain essential for dimerization and DNA binding. PTHS manifests with significant developmental delay, intellectual disability, limited speech, distinctive facial features, and breathing abnormalities (Pitt and Hopkins, 1978; Van Balkom et al., 2012; Teixeira et al., 2021).

Beyond its established role during development, a growing body of evidence suggests that TCF4 is also important for the mature brain’s function. In the adult brain, TCF4 is prominently expressed in the neocortex, hippocampus, cerebellum, and hypothalamic and amygdaloid nuclei, a pattern that is similar between humans and rodents (Rannals et al., 2016; Jung et al., 2018; Wang et al., 2020). The function of TCF4 in the mature brain remains poorly understood. Evidence suggests that it contributes to adult hippocampal neurogenesis and influences neuronal function and plasticity by regulating dendritic spine morphology, synaptic plasticity mechanisms such as long-term potentiation, neuronal excitability through ion channel gene regulation, and presynaptic function (Braun et al., 2020; Shariq et al., 2021; Crux et al., 2018). Through these functions, TCF4 may influence learning, memory, and sleep regulation.

The clinical importance of TCF4 in adulthood is highlighted by large-scale genetic studies that have identified variants within the *TCF4* locus as major risk factors for schizophrenia, bipolar disorder, major depression, and post-traumatic stress disorder (Del-Favero et al., 2002; Cross-Disorder Group of the Psychiatric Genomics, 2013; Wray et al., 2018; Gelernter et al., 2019; Teixeira et al., 2021). To understand the role of TCF4 in the mature brain and the cellular basis of these risks, it is essential to identify the specific cells that express TCF4. Here, we investigate the cell-type-specific expression of TCF4 in the neocortex, a primary site of pathology in these psychiatric disorders. We conducted this study in the macaque brain, which serves as a closer model to the human brain than rodent models.

## 2 Methods

### 2.1 Rhesus macaque tissue

No animals were sacrificed specifically for this study. Brain tissue from 8 rhesus macaques (*Macaca mulatta*) was obtained from the Amaral laboratory repository across the following developmental stages: gestation day (GD) 151 (*n =* 1, male), 14 days (*n =* 2, one female, one male), 1 month (*n =* 2, males), 3 months (*n =* 2, males), and 5 years (*n =* 1, male). All tissue was cryopreserved at −80 °C using established protocols (Rosene et al., 1986) that ensure long-term stability for immunohistochemical studies.

Details of animal preparation are provided in Burette et al. (2024) (see also Gonzalez Ramirez et al., 2024). Briefly, animals were deeply anesthetized with sodium pentobarbital (50 mg/kg i.v.) and transcardially perfused with 1% followed by 4% paraformaldehyde in 0.1 M phosphate buffer (PB, pH 7.4). Brains were minimally postfixed for 6 h, cryoprotected in graded glycerol/DMSO solutions, and frozen in isopentane. Perfusion parameters were adjusted for fetal cases. Brains were sectioned coronally at 30 μm in a 1:8 series using a freezing microtome, and the cryopreserved sections were stored at −80 °C.

### 2.2 Hybridization chain reaction fluorescent *in situ* hybridization

Hybridization chain reaction fluorescent *in situ* hybridization (HCR-FISH) labeling kits were purchased from Molecular Instruments (Pasadena, California), and experiments were performed according to the manufacturer’s instructions. Probe sets used in this study include Tcf4 (B3 initiator), Sox9 (B2), Vglut1 (B2), and Vgat (B4) and were detected using appropriate amplifier hairpins labeled with Alexa Fluor 488, 546, or 647. If needed, sections were then processed for immunofluorescence staining (see next section). Sections were examined with Leica STELLARIS 8 FALCON microscopes.

### 2.3 Immunohistochemistry

Free-floating sections were rinsed twice in phosphate buffered saline (PBS, 5 min each) and then in PBS containing 0.1% Triton X-100 (PBS-T). Sections were blocked for 30 min at room temperature in PBS-T with 10% fetal bovine serum. For experiments using primary antibodies from different host species, sections were incubated overnight with all primary antibodies (Table 1). After PBS-T rinses, sections were incubated with fluorescence-conjugated secondary antibodies and counterstained with DAPI and/or NeuroTrace (Invitrogen ThermoFisher Scientific, Waltham, MA). Tyramide signal amplification (TSA) was used to label both TCF4 and proCCK antibodies, which were raised in rabbit (Wang et al., 1999). Sections were first incubated with proCCK antibody at a dilution below the detection threshold for conventional secondary antibodies (1:20,000), but sufficient for TSA detection. Following overnight incubation, sections were processed with the TSA Kit (Biotium, Fremont, CA) according to the manufacturer’s instructions, after which conventional labeling procedures were performed. Mounted sections were air-dried and coverslipped with Vectashield Plus (Vector Laboratories, H-1900). Images were acquired on a Leica STELLARIS 8 FALCON microscope.

**Table 1:** Details of antibodies used in this study.

Target protein	Primary antibody	Immunogen	Secondary antibody
Calretinin	Goat antibody Swant Cat# CG1 RRID: AB_10000342 Concentration: 1:2,000	Human recombinant calretinin	Donkey anti-Goat IgG Alexa Fluor Plus 555 Thermo Fisher Scientific Cat# A32816 RRID: AB_2762839 Concentration: 1:400
GAD	Mouse monoclonal Clone ID: 1G10.2 Millipore Cat# MAB5406 RRID: AB_2278725 Concentration: 1:1,000	Recombinant GAD67 protein	Donkey anti-Mouse IgG Alexa Fluor 568 Jackson ImmunoResearch Labs Cat# 715–575-151 RRID: AB_3095482 Concentration: 1:400
GFAP	Mouse monoclonal Clone ID: G-A-5 Sigma-Aldrich Cat# G6171 RRID: AB_1840893 Concentration: 1:2,000	Purified GFAP from pig spinal cord	Donkey anti-Mouse IgG Alexa Fluor 647 Jackson ImmunoResearch Labs Cat# 715–605-151 RRID: AB_2340863 Concentration: 1:400
NeuN	Guinea pig polyclonal Millipore Cat# ABN90 RRID: AB_11205592 Lot: 3854129 Concentration: 1:1,000	GST-tagged recombinant fragment corresponding to the first 97 amino acids of mouse NeuN.	Donkey anti-Guinea pig IgG Alexa Fluor 647 Jackson ImmunoResearch Labs Cat# 706–606-148 RRID: AB_2340477 Concentration: 1:400
Olig 2	Mouse monoclonal Clone ID: 211F1.1 GeneTex Cat# GTX01779 Concentration: 1:500	Recombinant protein corresponding to human OLIG2.	Donkey anti-Mouse IgG Alexa Fluor 568 Jackson ImmunoResearch Labs Cat# 715–575-151 RRID: AB_3095482 Concentration: 1:400
Parvalbumin	Mouse monoclonal Swant Cat#: PV235 RRID: AB_3698492 Concentration: 1:2,000	-	Donkey anti-Mouse IgG Alexa Fluor Plus 594 Thermo Fisher Scientific Cat# A-21203 RRID: AB_2535789 Concentration: 1:400
proCCK	Rabbit polyclonal Frontier Institute Cat# CCK-pro-Rb RRID: AB_2571674 Concentration: 1:20,000	Mouse CCK, 107-115aa	Tyramide Amplification with HRP Streptavidin (biotium, Cat# 33920), followed by Alexa Fluor 790 Streptavidin (Jackson ImmunoResearch Labs, 016–650-084)
Somatostatin	Rat monoclonal Clone ID: SY-160F7 Synaptic Systems Cat# 366017 RRID: AB_3083022 Concentration: 1:1,000	Mouse somatostatin, 89-100aa	Donkey anti-Rat IgG Alexa Fluor Plus 647 Thermo Fisher Scientific Cat# A48272TR RRID: AB_2896338 Concentration: 1:400
SOX9	Goat polyclonal R and D Systems Cat# AF3075 RRID: AB_2194160 Concentration: 1:1,000	Human SOX9: Met1-Lys151	Donkey anti-Goat IgG Alexa Fluor 568 Jackson ImmunoResearch Labs Cat# 705–575-147 RRID: AB_3095466 Concentration: 1:400
TCF4	Rabbit monoclonal Clone ID: NCI-R159-6 Abcam ab217668 RRID: AB_2714172 Concentration: 1:800	Central region^1^	Donkey anti-Rabbit IgG Alexa Fluor Plus 488 Thermo Fisher Scientific Cat# A32790TR RRID: AB_2866495 Concentration: 1:400
TMEM119	Mouse monoclonal Clone ID: CL8714 Atlas Antibodies AMAb91528 RRID: AB_2797214 Concentration: 1:10,000	Human recombinant: GDGARMVEGR GAEEEEKGSQ EGDQEVQGHG VPVETPEAQE EPCSGVLEGA VVAGEGQGEL EGSLLLAQEA QGPVGPPESP CACSSVHPS	Donkey anti-Mouse IgG Alexa Fluor 568 Jackson ImmunoResearch Labs Cat# 715–575-151 RRID: AB_3095482 Concentration: 1:400

GAD, Glutamic acid decarboxylase; GFAP, Glial fibrillary acidic protein; OLIG2, Oligodendrocyte transcription factor 2; proCCK, pro-cholecystokinin; TCF4, Transcription factor 4; TMEM119, Transmembrane protein 119. ^1^The exact immunogen sequence is proprietary. Abcam has confirmed that it targets a “central” region of the protein (i.e., not at the N or C terminal part), enabling the antibody to react with all TCF4 isoforms in mice, macaques, and humans (personal communication from Abcam to ACB).

### 2.4 Quantification

Cell and nuclear segmentation were performed using InstanSeg, a deep learning framework optimized for multiplexed fluorescence microscopy, implemented within QuPath (v0.5, RRID: SCR_018257). The pre-trained fluorescence model generates per-pixel embeddings that cluster pixels by individual cell identity while maintaining separation between neighboring cells, producing reliable masks. Only cells with both nucleus and cytoplasm fully detected were included in the analysis. Three sections per animal were analyzed. For each experimental set, confocal acquisition settings were optimized and maintained consistently across all sections and ages within that experiment.

Raw nuclear and cytoplasmic intensities for all labeling were computed for each cell and exported to OriginPro 2024b (OriginLab, Northampton, MA) for further analysis. Cell-type identity was assigned using marker-specific intensity thresholds: nuclear intensity for SOX9, combined nuclear and cytoplasmic intensity for NeuN, GAD, somatostatin, calretinin, and parvalbumin, and cytoplasmic intensity for CCK. Thresholds were set manually, guided by the bimodal distribution of staining intensities and confirmed by visual inspection. Because the markers used in this study exhibit a clear on/off expression pattern, threshold selection was straightforward. Thresholds were set conservatively, retaining only cells that were unambiguously marker-positive. Quantification could not be performed blind to developmental age, as animal age was readily apparent from tissue morphology.

Raw nuclear TCF4 intensities were normalized to a 0–1 scale using min-max normalization across all sections included in each analysis — either multiple sections from the same animal, or across animals of different ages the case of Figure 9. To facilitate descriptive comparison across cell types and developmental stages, normalized intensities were referenced to the section-level mean (x̄) and standard deviation (*σ*), and cells were assigned to one of four categories: negative-to-weak (< x̄), average (x̄ to x̄+σ), intense (x̄+σ to x̄+2σ), or very intense (> x̄+2σ).

### 2.5 Re-analysis of public transcriptomic datasets

#### 2.5.1 Bulk-tissue developmental atlases

Two published developmental brain transcriptomic atlases were used to characterize TCF4 expression across different brain regions, developmental periods, and species. The Kang et al. (2011) dataset was obtained from the NCBI Gene Expression Omnibus (accession GSE25219). This dataset provides measurements from the Affymetrix GeneChip Human Exon 1.0 ST array for 1,340 brain tissue samples from 16 brain regions of 57 postmortem human donors, covering 15 developmental periods (P1-P15), spanning early embryonic development through middle adulthood. The transcript-level expression matrix (platform GPL5175; 17,565 transcript clusters; RMA-normalized log2 intensities) was downloaded using the GEOquery Bioconductor package (v2.78). TCF4 was identified by Affymetrix transcript cluster ID 3808854, mapped through the huex10sttranscriptcluster.db annotation package (v8.8). Median TCF4 log2 expression across all samples was 11.05 (range 8.26–12.74), significantly above the array background.

The Zhu et al. (2018) dataset was obtained from the Human Brain Evolution portal.^1^ This dataset comprises 460 human samples from 36 donors and 366 rhesus macaque samples from 26 donors, covering 16 brain regions across prenatal and postnatal development. TCF4 was identified via Ensembl ID ENSG00000196628.14. RPKM values were transformed to log2 [max (RPKM, 0.1)] for downstream visualization.

To ensure consistency across the two bulk-tissue datasets, we restricted our analysis to 16 anatomically matched brain regions: 11 neocortical areas (orbital prefrontal cortex, dorsolateral prefrontal cortex, ventrolateral prefrontal cortex, medial prefrontal cortex, primary motor cortex, primary somatosensory cortex, posterior inferior parietal cortex, primary auditory cortex, superior temporal cortex, inferolateral temporal cortex, and primary visual cortex), 4 subcortical regions (hippocampus, amygdala, striatum, and mediodorsal nucleus of the thalamus), and the cerebellar cortex. The analysis cohort comprised 1,281 Kang et al. (2011) human samples from 56 donors, 439 Zhu et al. (2018) human samples from 36 donors, and 366 Zhu et al. (2018) macaque samples from 26 donors. Sample developmental ages were mapped onto the 15 developmental periods defined for human development by Kang et al. (2011): P1, embryonic (4–8 postconceptional weeks [pcw]); P2, early fetal (8–10 pcw); P3, early fetal (10–13 pcw); P4, early mid-fetal (13–16 pcw); P5, early mid-fetal (16–19 pcw); P6, late mid-fetal (19–24 pcw); P7, late fetal (24–38 pcw); P8, neonatal (birth–6 months); P9, early infancy (6–12 months); P10, late infancy (1–3 years); P11, early childhood (3–6 years); P12, middle–late childhood (6–12 years); P13, adolescence (12–20 years); P14, young adulthood (20–40 years); and P15, middle adulthood (40–60 years). For the Zhu et al. (2018) dataset, the authors’ precomputed predicted period harmonization was used, which maps macaque developmental ages onto the Kang et al. (2011) human period scale. All bulk-atlas analyses were performed in R 4.5.3 using GEOquery, Biobase, AnnotationDbi, data.table, ggplot2, and patchwork.

#### 2.5.2 Single-nucleus RNA-seq data mining

To evaluate TCF4 expression at a cellular resolution, we analyzed single-nucleus RNA-sequencing (snRNA-seq) data from the adult rhesus macaque brain (Chiou et al., 2023), accessed via the CELLxGENE Discover portal. Data were derived from three-level single-nucleus combinatorial-indexing RNA-seq (sci-RNA-seq3) profiling of four 10-year-old male rhesus macaques. We utilized two complementary subsets: a brain-wide atlas (1.5 million nuclei across 30 regions and 17 cell classes) and a focused GABAergic-neuron dataset (371,548 nuclei). TCF4 transcripts were identified using the *Macaca mulatta* Ensembl ID ENSMMUG00000012001.

For the brain-wide analysis, per-nucleus mRNA abundance was extracted from the 1.5 million-cell matrix. These data represent library-size-normalized, log-transformed counts, with each value reflecting TCF4 sequencing reads from an individual nucleus, corrected for total sequencing depth. Each nucleus was cross-referenced with its anatomical region and cell-class annotation from the original metadata. To identify enrichment patterns, we calculated the average per-nucleus value for each of the 30 regions × 17 cell classes. This included all nuclei, even those with zero detected TCF4 transcripts. The resulting 30 × 17 mean-expression matrix was visualized as a heatmap to reveal cell-type-specific and regional expression patterns.

For the comparison of cortical GABAergic versus glutamatergic cells, only those derived from the three cortical regions (frontal, parietal, temporal) were included and labeled as either GABAergic or glutamatergic neurons.

For the interneuron-subtype analysis, data from a 371,548-cell GABAergic subset was used. Since the deposited cell subcluster labels are numbered generically (“GABAergic neurons 1” through “GABAergic neurons 20”) without specific subtype names, each subcluster was identified anew by its marker-gene signature. Per-cell expression of seven canonical markers was extracted: the medial ganglionic eminence (MGE)/caudal ganglionic eminence (CGE) lineage transcription factors LIM homeobox 6 (LHX6), and the subtype markers parvalbumin (PV), somatostatin (SST), calbindin 2 (i.e., calretinin, CR), vasoactive intestinal peptide (VIP), cholecystokinin (CCK), and lysosomal-associated membrane protein 5 (LAMP5). The mean expression of each marker within each subcluster was calculated, and the six candidate subtype markers were column-z-scored across subclusters to identify the most enriched marker in each. Each subcluster was then assigned to a subtype based on its top z-scored marker, provided the top z-score was >0.3. Subclusters that did not meet this threshold were labeled “Mixed / LHX6.” The analysis was limited to cells from the four neocortical regions, resulting in 273,253 neocortical interneuron nuclei. All single-nucleus analyses were conducted in Python 3.12 using anndata (v0.12.10) and scanpy (v1.12).

## 3 Results

### 3.1 TCF4 expression in the young adult macaque neocortex is predominantly neuronal

**Figure 1:**
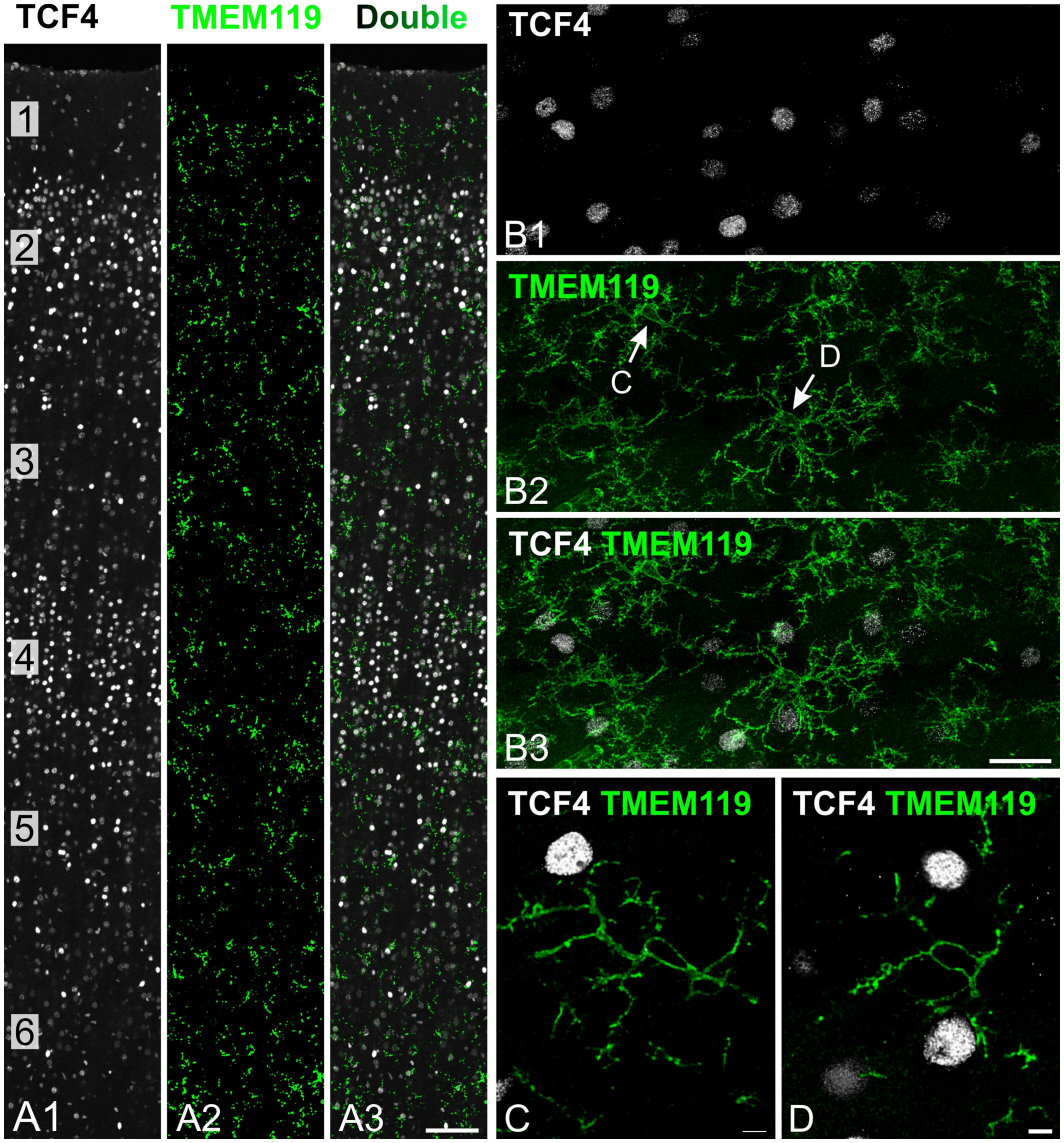
TCF4 immunostaining is not detected in microglia in the young adult macaque neocortex. Immunofluorescence staining of temporal cortex from a 5-year-old macaque showing TCF4 (white) and TMEM119 (green) immunostaining. **(A1–A3)** Overview of cortical layers 1–6 showing nuclear TCF4 staining concentrated in layers 2 and 4, with TMEM119-positive microglia uniformly distributed across all layers. **(B1–B3)** Higher-magnification views of layers 2/3 show strictly nuclear TCF4 localization and TMEM119-positive microglia displaying ramified morphology characteristic of resting microglia. Arrows indicate microglia shown at high resolution in panels **(C,D)**. **(C,D)** High-magnification images of individual TMEM119-positive microglia showing the absence of detectable TCF4 staining in these cells. Scale bars: **(A)** 100 μm; **(B)** 25 μm; **(C,D)** 5 μm.

We examined TCF4 expression in non-neuronal cells within the neocortex of a five-year-old macaque. Consistent with previous reports, TCF4 immunostaining was exclusively nuclear, with staining intensity varying from weak to intense (Figure 1) (Burette et al., 2024). Intensely labeled nuclei were distributed across all cortical layers but showed notable concentration in layers 2 and 4 (Figure 1A). This characteristic bilayer distribution pattern was most prominent in the temporal cortex and absent in the motor cortex, which has a much-reduced layer 4. To avoid unnecessary repetition, we focus our narrative on the temporal cortex from this point forward, as we observed no notable differences in TCF4 expression patterns across cell types in other neocortical regions (e.g., somatosensory, motor, and visual cortices).

To examine TCF4 expression in microglia, we used TMEM119, a type I transmembrane protein that selectively labels resident microglia in their homeostatic state but does not mark peripheral macrophages (Bennett et al., 2016; Vankriekelsvenne et al., 2022). TMEM119-labeled cells were distributed uniformly throughout all cortical layers (Figure 1A). They exhibited the characteristic ramified morphology typical of microglia in their resting or surveillance state (Vidal-Itriago et al., 2022) (Figure 1A2). We did not detect TCF4 immunoreactivity in TMEM119-positive cells, suggesting that non-activated microglia have negligible or absent TCF4 protein.

**Figure 2:**
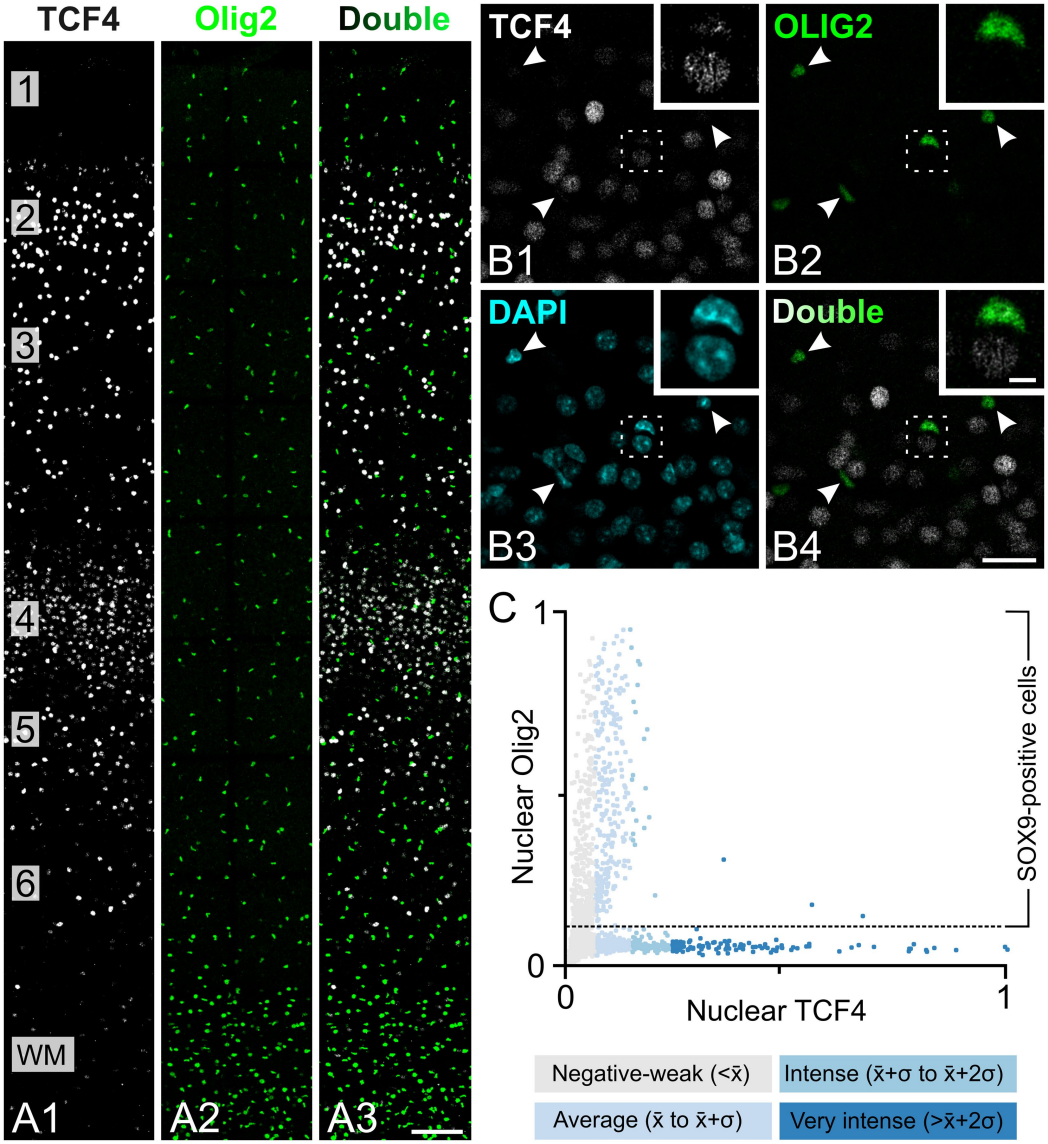
Oligodendrocytes show low TCF4 immunostaining in the young adult macaque neocortex. Triple immunofluorescence staining of temporal cortex from a 5-year-old macaque for TCF4 (white), OLIG2 (green), and DAPI (cyan). **(A1–A3)** Overview of cortical layers 1–6 showing nuclear TCF4 staining concentrated in layers 2 and 4, with OLIG2-positive oligodendrocytes uniformly distributed across all layers and enriched in the white matter (WM). **(B)** Field from layer 2 showing OLIG2-positive oligodendrocytes (arrowheads) displaying weak-to-moderate TCF4 immunoreactivity, while strongly TCF4-positive nuclei are OLIG2-negative. The inset shows a higher magnification of the boxed area. **(C)** A scatter plot showing OLIG2 versus TCF4 nuclear immunoreactivity intensity shows that oligodendrocytes predominantly exhibit negative-to-weak (<x̄) or average (x̄ to x̄+*σ*) TCF4 expression levels, while cells with intense or very intense TCF4 labeling are OLIG2-negative. Scale bars: **(A)** 25 μm; insets: 5 μm.

Next, we investigated TCF4 expression in oligodendrocytes. We performed triple immunolabeling for TCF4, OLIG2 (a transcription factor expressed in oligodendrocytes and their precursors), and DAPI (nuclear label). OLIG2-positive nuclei were uniformly distributed across all cortical layers and showed enrichment in the white matter (Figure 2A). Most OLIG2-positive nuclei exhibited weak TCF4 immunoreactivity (Figure 2B). Quantitative analysis showed that OLIG2-positive cells displayed either negative-to-weak TCF4 intensity (defined as below average, < x̄) or average TCF4 intensity (defined between the mean and one standard deviation above the mean, x̄ to x̄+*σ*) (Figure 2C). Cells exhibiting intense TCF4 labeling (defined as between one and two standard deviations above average, x̄+σ to x̄+2σ) or very intense TCF4 labeling (defined as more than two standard deviations above average, > x̄+2σ) were not positive for OLIG2.

**Figure 3:**
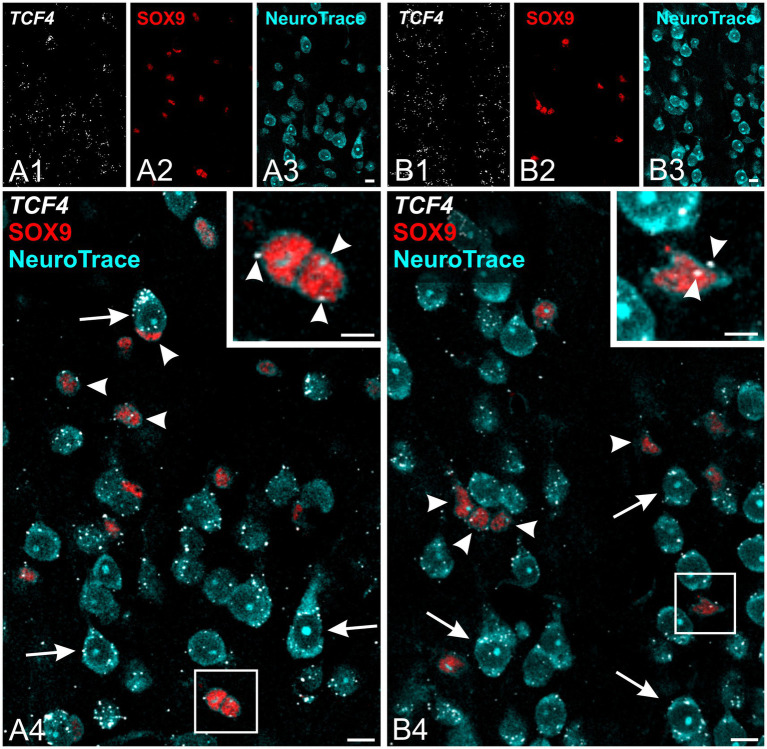
Astrocytes show lower *TCF4* mRNA expression compared to neurons in the young adult macaque neocortex. Fluorescent *in situ* hybridization for *TCF4* mRNA (white) combined with immunofluorescence staining for SOX9 (red; astrocyte marker) and NeuroTrace stain (cyan; broadly labeling cell cytoplasm and nucleoli) in the temporal cortex from a 5-year-old macaque. Fields in layers 1/2 **(A)** and layer 4 **(B)** showing SOX9-positive astrocytes (arrowheads) with weaker *TCF4* labeling compared to probable neurons (arrows). Insets are higher magnification of boxed areas in A and B showing SOX9-positive astrocytes with low *TCF4* expression; arrowheads point to *TCF4* mRNA labeling. Scale bars: **(A,B)** 10 μm; insets: 5 μm.

**Figure 4:**
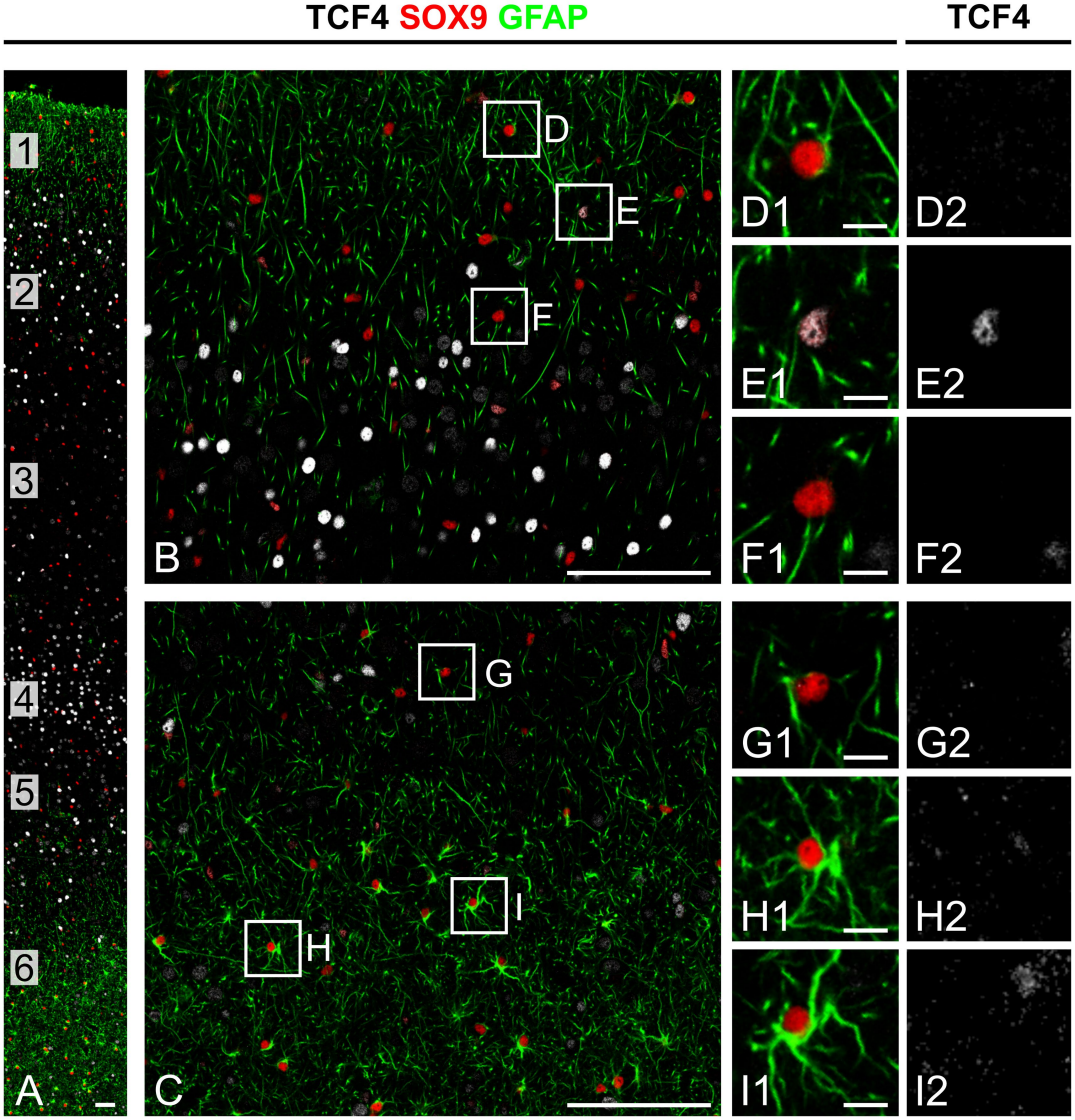
Astrocytes show low TCF4 immunostaining in the young adult macaque neocortex. Triple immunofluorescence staining of temporal cortex from a 5-year-old macaque for TCF4 (white), SOX9 (red; marker for all astrocytes), and GFAP (green; commonly used astrocyte marker). **(A)** Low-magnification image across cortical layers 1–6. While SOX9-positive astrocytes are uniformly distributed across all layers, GFAP-positive astrocytes concentrate in superficial layers (1 and 2) and deep layers (5 and 6). **(B,C)** Higher-magnification views of superficial **(B)** and deep **(C)** cortical regions. **(D–I)** High-magnification views of boxed areas in **(B,C)**. Most SOX9-positive cells, regardless of GFAP positivity, show weak TCF4 immunostaining, with only rare exceptions as seen in **(E)**. Scale bars: **(A)** 50 μm; **(B,C)** 100 μm; **(D**–**I)** 10 μm.

**Figure 5:**
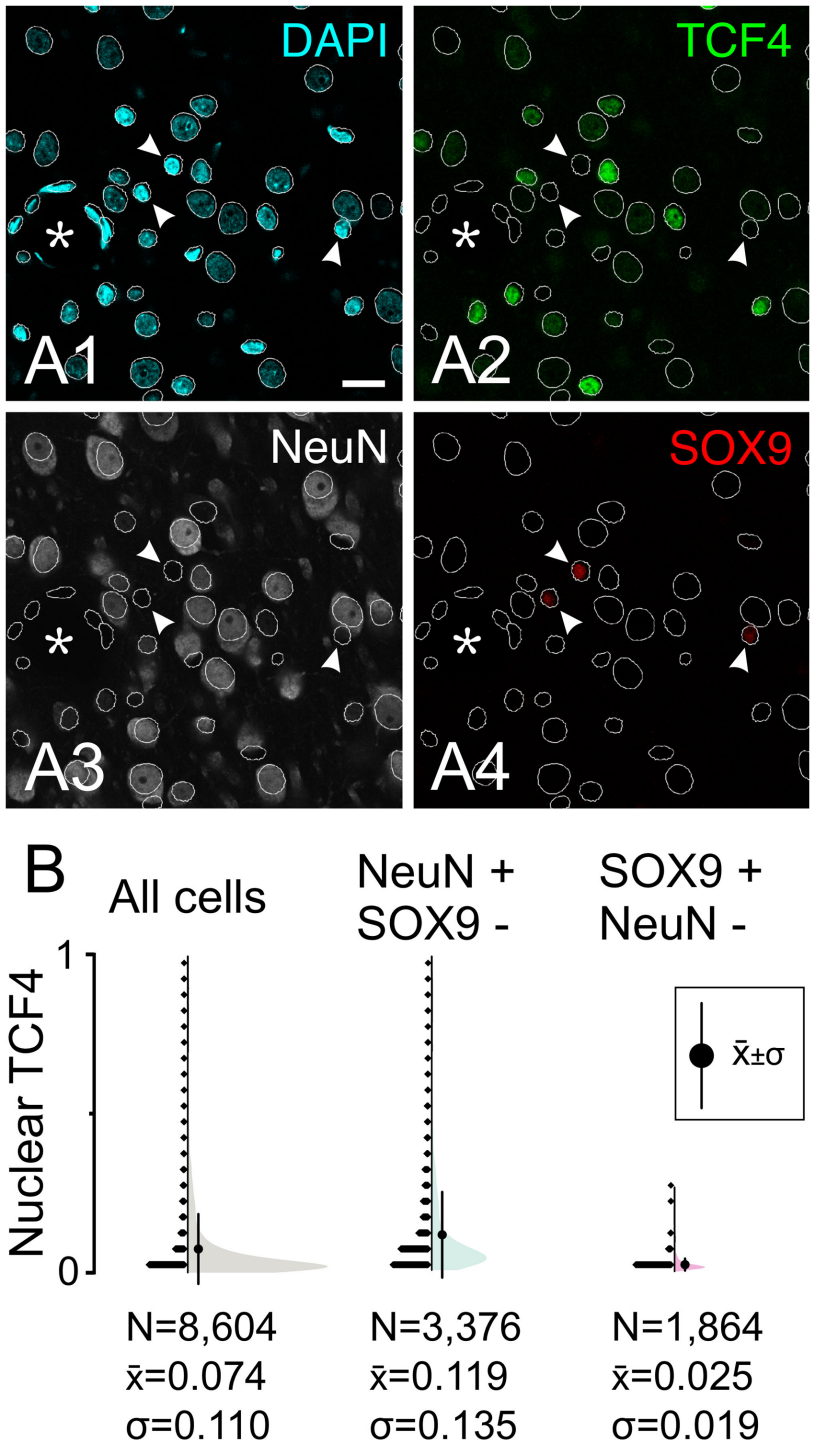
TCF4 is enriched in neurons in the young adult macaque neocortex. Quadruple immunofluorescence staining of temporal cortex from a 5-year-old macaque for TCF4 (green), NeuN (white), SOX9 (red), and DAPI (cyan). **(A)** Field from layer 2 showing SOX9-positive astrocytes (arrowheads) with low TCF4 immunoreactivity. All DAPI-stained nuclei are automatically identified and outlined (white contours), including nuclei from probable blood vessels (asterisk), demonstrating the effectiveness of the quantitative analysis methods. **(B)** Quantitative analysis of nuclear TCF4 immunostaining intensity across cell populations. Neurons (NeuN-positive/SOX9-negative) show higher TCF4 immunostaining compared to astrocytes (SOX9-positive/NeuN-negative) and the general cell population (all detected nuclei). The higher standard deviation in neurons reflects heterogeneous TCF4 expression levels, while astrocytes show consistently low expression. Values represent normalized nuclear TCF4 intensity relative to the overall cell population (e.g., all detected DAPI nuclei). Scale bar: 20 μm.

We next examined TCF4 expression in astrocytes using *in situ* hybridization combined with immunofluorescence staining for SOX9, a pan-astrocyte marker. SOX9-positive astrocytes showed weaker *TCF4* mRNA expression compared to neurons, which were identified by their larger size and distinctive morphology with NeuroTrace staining (Figure 3). To evaluate TCF4 protein levels in astrocytes, we conducted quintuple immunolabeling for TCF4, SOX9, GFAP, NeuN, and DAPI (Figure 4). SOX9-positive/GFAP-negative astrocytes were distributed uniformly across all cortical layers (Figure 4A). By contrast, SOX9-positive/GFAP-positive astrocytes—which provide structural support and maintain blood–brain barrier integrity—were primarily localized to superficial (layers 1 and 2) and deep (layers 5 and 6) cortical layers, the subpial region, the vicinity of major blood vessels, and adjacent white matter. Qualitative assessment showed consistently low TCF4 protein expression in SOX9-positive nuclei across all examined ages, regardless of GFAP co-expression status (Figures 4B–I). Only a small subpopulation of SOX9-positive/GFAP-negative astrocytes exhibited high TCF4 levels (see Figure 4E). Quantitative analysis confirmed the visual inspection. The results showed SOX9-positive/NeuN-negative cells have a lower TCF4 signal than the general cell population, whereas NeuN-positive/SOX9-negative cells have the highest signal (Figure 5).

### 3.2 A postnatal transition establishes high TCF4 expression in GABAergic neurons

**Figure 6:**
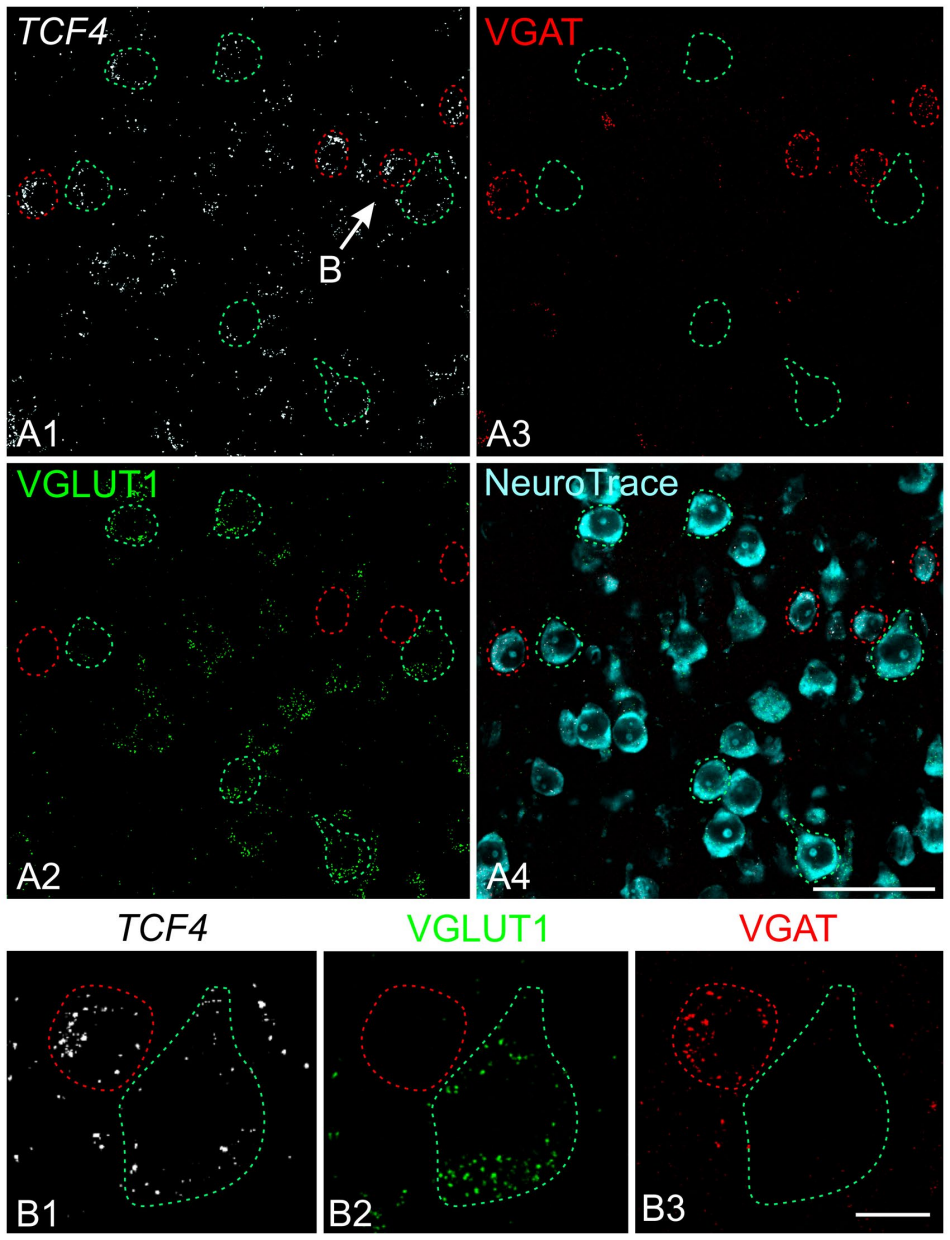
*TCF4* mRNA is expressed in both excitatory and inhibitory neurons. Multiplex fluorescent *in situ* hybridization for *TCF4* (white), VGLUT1 (green; excitatory neuron marker), VGAT (red; inhibitory neuron marker), and NeuroTrace stain (cyan). **(A)** Outlined neurons show *TCF4* labeling in excitatory (expressing VGLUT1 but not VGAT, outlined in green) and inhibitory cells (expressing VGAT but not VGLUT1, outlined in red). **(B)** High-magnification view of the region indicated by the arrow in **(A1)**, showing *TCF4* mRNA expression in individual neurons in more detail. Scale bars: **(A)** 50 μm; **(B)** 10 μm.

To investigate the population of neurons expressing high TCF4 levels, we began by using multiplex *in situ* hybridization in the young adult neocortex. We detected *TCF4* mRNA in both excitatory (VGLUT1-positive) and inhibitory (VGAT-positive) neurons (Figure 6). The signal intensity varied significantly from cell to cell, with inhibitory neurons generally exhibiting more *TCF4* labeling than excitatory neurons.

**Figure 7:**
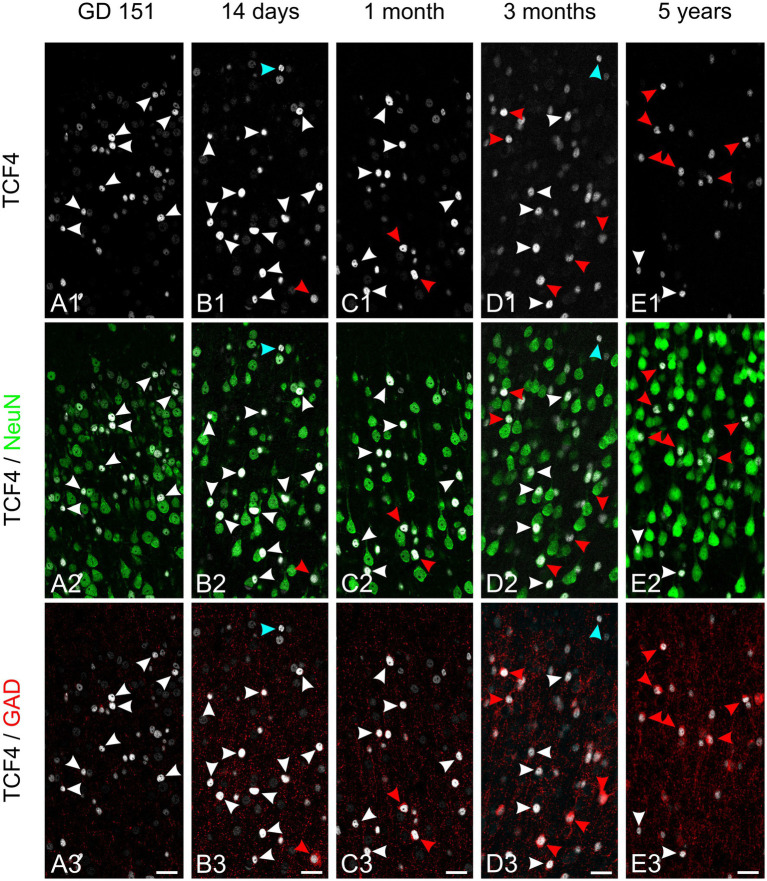
Developmental transition of TCF4 immunoreactivity in superficial cortical layers. Triple immunofluorescence staining of superficial cortical layers (layers 2/3) in temporal cortex for TCF4 (white), NeuN (green), and GAD (red) across five developmental stages: GD 151 **(A)**, 14 days **(B)**, 1 month **(C)**, 3 months **(D)**, and 5 years **(E)**. White arrowheads point to probable excitatory neurons (NeuN-positive but GAD-negative) and red arrowheads point to GABAergic neurons (NeuN- and GAD-positive). At GD 151 and early neonatal stages, high TCF4 immunostaining is observed in both excitatory and inhibitory neurons. As maturation progresses, high TCF4 immunostaining becomes more restricted to GABAergic neurons. Blue arrowhead in D3 indicates a rare non-neuronal cell with high TCF4 immunostaining. Scale bars: 50 μm.

**Figure 8:**
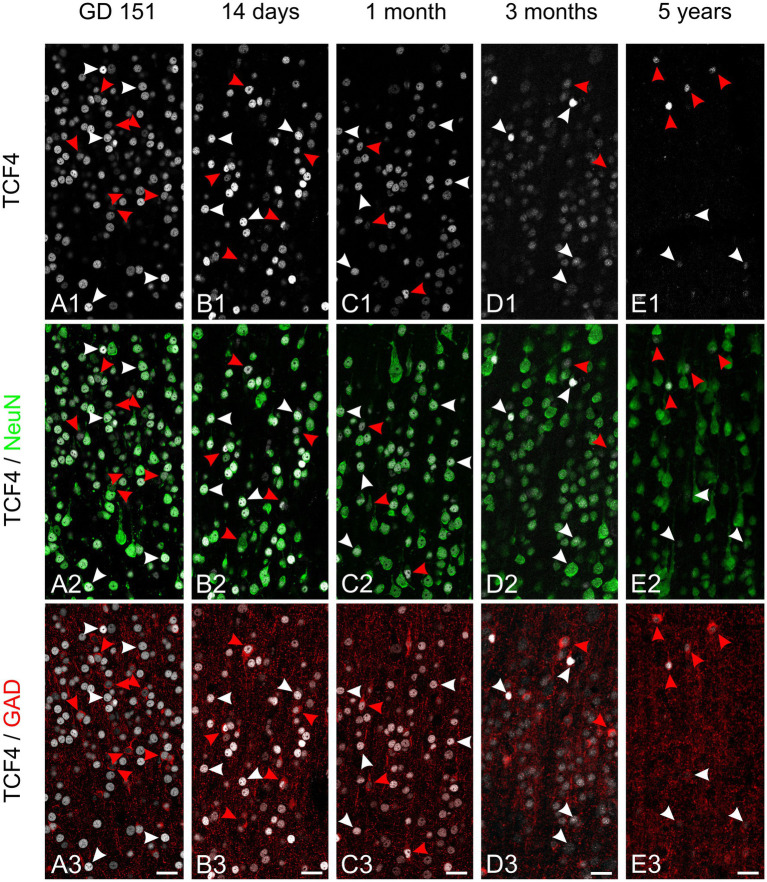
Developmental transition of TCF4 expression in deep cortical layers. Triple immunofluorescence staining of deep cortical layers (layer 4) in temporal cortex for TCF4 (white), NeuN (green), and GAD (red) across five developmental stages: GD 151 **(A)**, 14 days **(B)**, 1 month **(C)**, 3 months **(D)**, and 5 years **(E)**. White arrowheads indicate probable excitatory neurons (NeuN-positive but GAD-negative) and red arrowheads indicate GABAergic neurons (NeuN- and GAD-positive). At GD 151, most excitatory neurons show high TCF4 expression, while most GABAergic neurons display lower immunostaining. As maturation progresses, similar to superficial layers, high TCF4 immunostaining becomes more restricted to GABAergic neurons. Values represent normalized nuclear TCF4 intensity relative to the overall cell population (e.g., all detected DAPI nuclei) across all ages. Scale bars: 50 μm.

We next tracked TCF4 expression in excitatory and inhibitory neurons across development by analyzing neocortex from late gestation (GD 151), neonatal (2 and 4 weeks old), infant (3 months), and young adult (5.5 years) macaques. We performed quadruple immunolabeling for TCF4, NeuN, GAD, and DAPI (Figures 7, 8). Initial examination of TCF4 and NeuN labeling confirmed highly variable TCF4 expression in neurons (Figures 7, 8). Across all examined ages, TCF4 was concentrated in layers 2 and 4, with neurons displaying a wide range of labeling intensities. From GD 151 to 5 years of age, overall TCF4 levels decreased.

The temporal–spatial development pattern of the neocortex allowed for the visualization of the maturational process within individual sections, with the superficial layers (Figure 7) being less mature than the deeper layers (Figure 8). At GD 151, we observed minimal GAD-positive neurons in superficial layers; consequently, most intensely TCF4-labeled neurons were NeuN-positive and GAD-negative. From 2 weeks to 3 months, the number of GAD-positive cells increased, with many exhibiting high TCF4 levels. While many NeuN-positive/GAD-negative cells also showed high TCF4 levels, most displayed low to moderate expression. By 5 years, cells with high TCF4 expression were predominantly GAD-positive.

In layer 4 at GD 151, GAD-positive neurons were common. Still, they expressed only low to moderate TCF4 levels, with intensely labeled neurons being GAD-negative (Figure 8). From 2 weeks to 3 months, the number of TCF4-labeled GAD-positive cells increased. By 5 years, the most strongly labeled cells were also GAD-positive.

**Figure 9:**
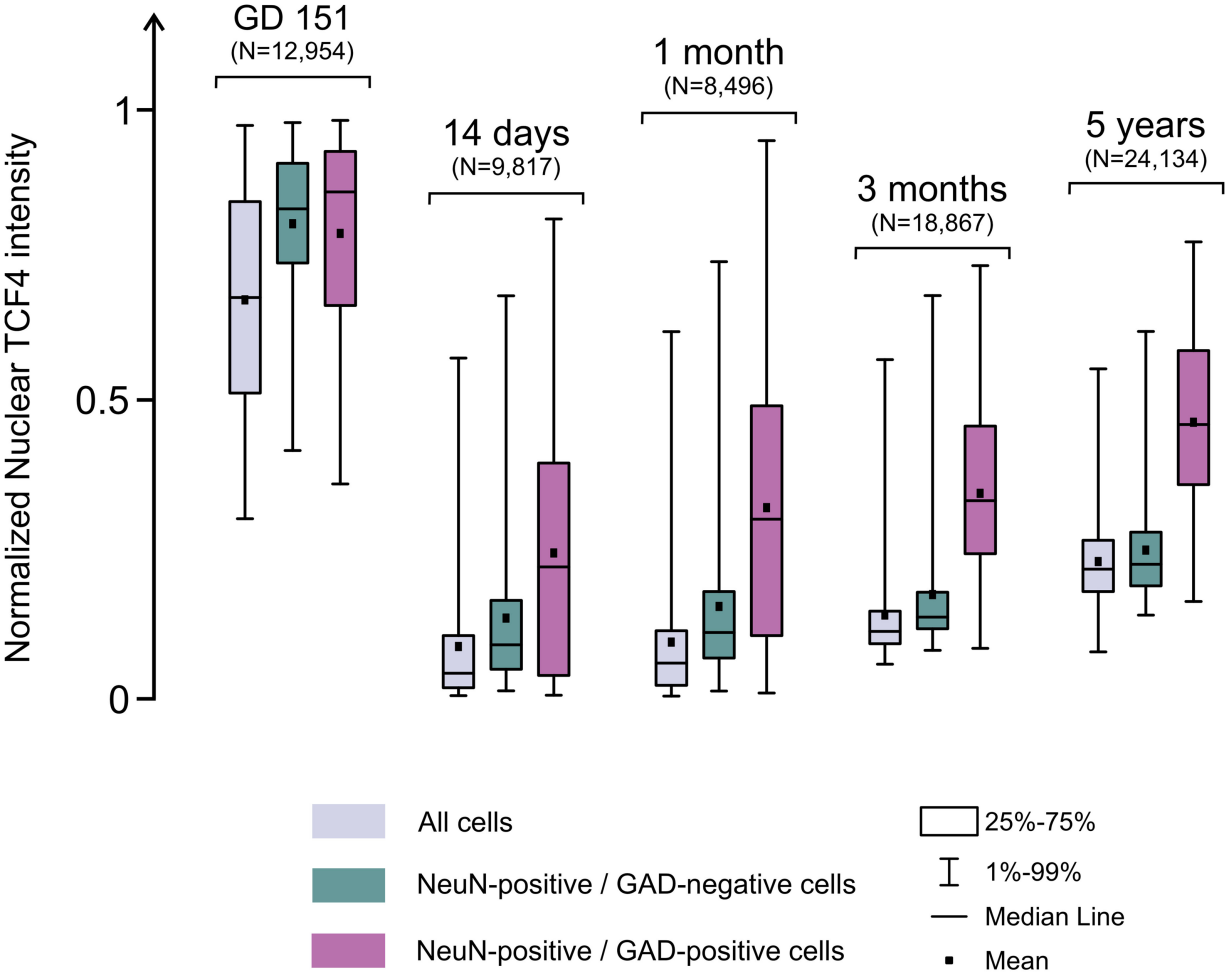
Developmental dynamics of nuclear TCF4 expression in excitatory and inhibitory neurons. Box plots showing nuclear TCF4 intensity in the neocortex at GD 151 (*n =* 1), 14 days (*n =* 2), 1 month (*n =* 2), 3 months (*n =* 2), and 5 years (*n =* 1). Data are shown for all cells (gray), NeuN-positive/GAD-negative (excitatory neurons, teal), and NeuN-positive/GAD-positive (inhibitory neurons, magenta). At GD 151, both excitatory and inhibitory neurons show elevated TCF4 expression above the general cell population. With advancing maturation, TCF4 expression in excitatory neurons progressively decreases toward levels similar to the general population, while inhibitory neurons maintain consistently higher expression than excitatory neurons.

Quantification of nuclear TCF4 labeling showed that TCF4 expression decreased dramatically from GD 151 to 2 weeks. At GD 151, neurons, regardless of GAD expression, showed high TCF4 levels exceeding the general cell population (Figure 9). At 2 weeks, neurons still exhibited higher TCF4 expression than the general cell population, with GAD-positive neurons expressing more TCF4 than GAD-negative neurons. From 2 weeks to 5 years, TCF4 expression in GAD-negative neurons approached levels similar to the general cell population, while GAD-positive neurons maintained higher expression than GAD-negative neurons.

Additionally, at 2 weeks, GAD-positive neurons displayed wide variability in TCF4 expression, which decreased during neocortical maturation. At 5 years, though reduced, substantial variability persisted, suggesting potential differential TCF4 expression across GABAergic neuron subtypes.

### 3.3 TCF4 expression varies significantly across GABAergic neuron subtypes

**Figure 10:**
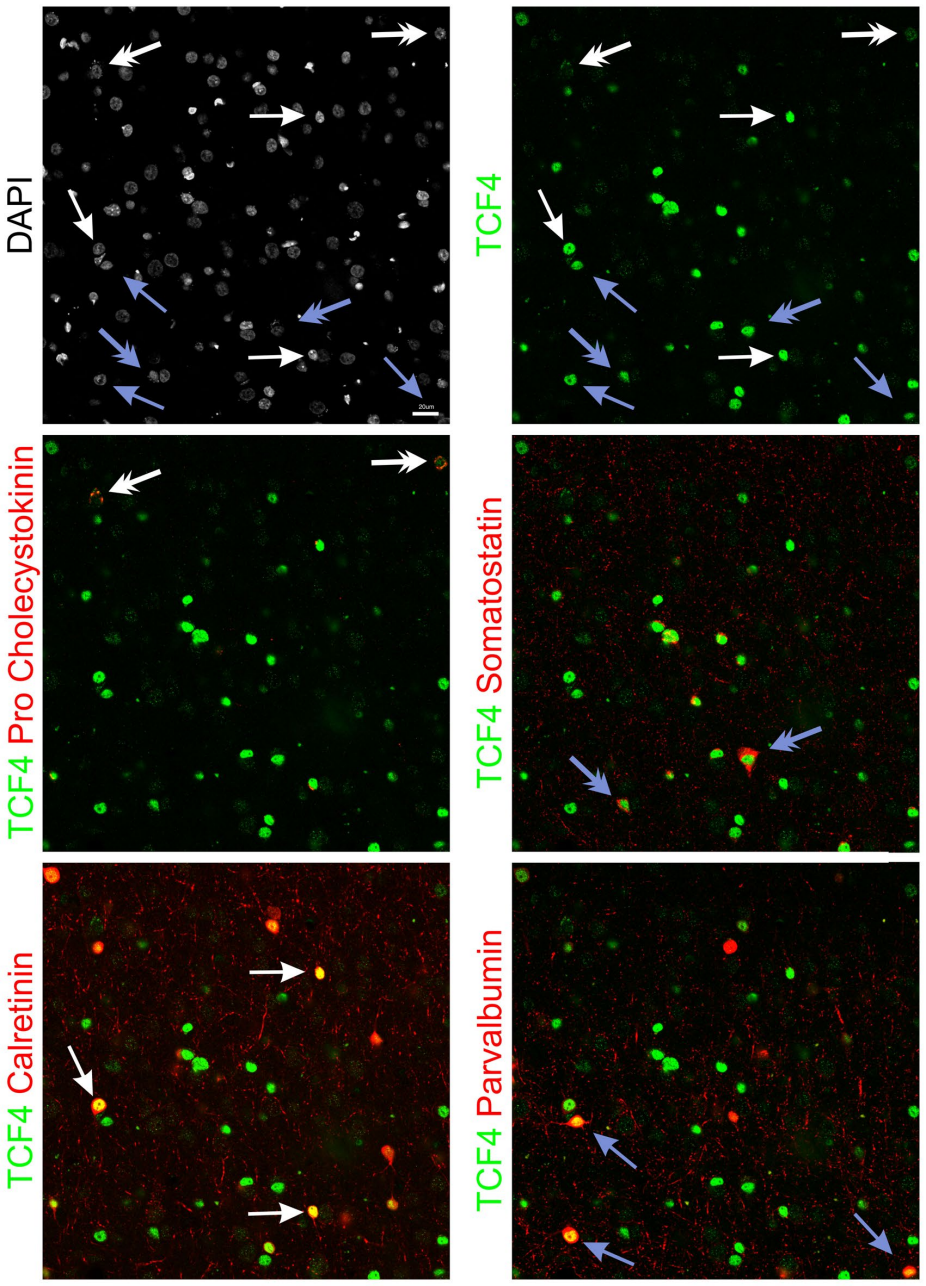
TCF4 immunostaining across GABAergic interneuron subtypes in the young adult macaque neocortex. Sextuple immunofluorescence staining of temporal cortex from a 5-year-old male macaque showing TCF4 (green) with markers for major interneuron subtypes: pro-cholecystokinin, somatostatin, calretinin, and parvalbumin, all shown in red, along with DAPI (gray). Each panel shows TCF4 expression with a different interneuron marker. White arrows indicate cells positive for calretinin; blue arrows indicate cells positive for parvalbumin; white double arrows indicate cells positive for pro-cholecystokinin; blue double arrows indicate cells positive for somatostatin. TCF4 immunoreactivity is present across all interneuron subtypes with varying intensities. Scale bar: 20 μm.

**Figure 11:**
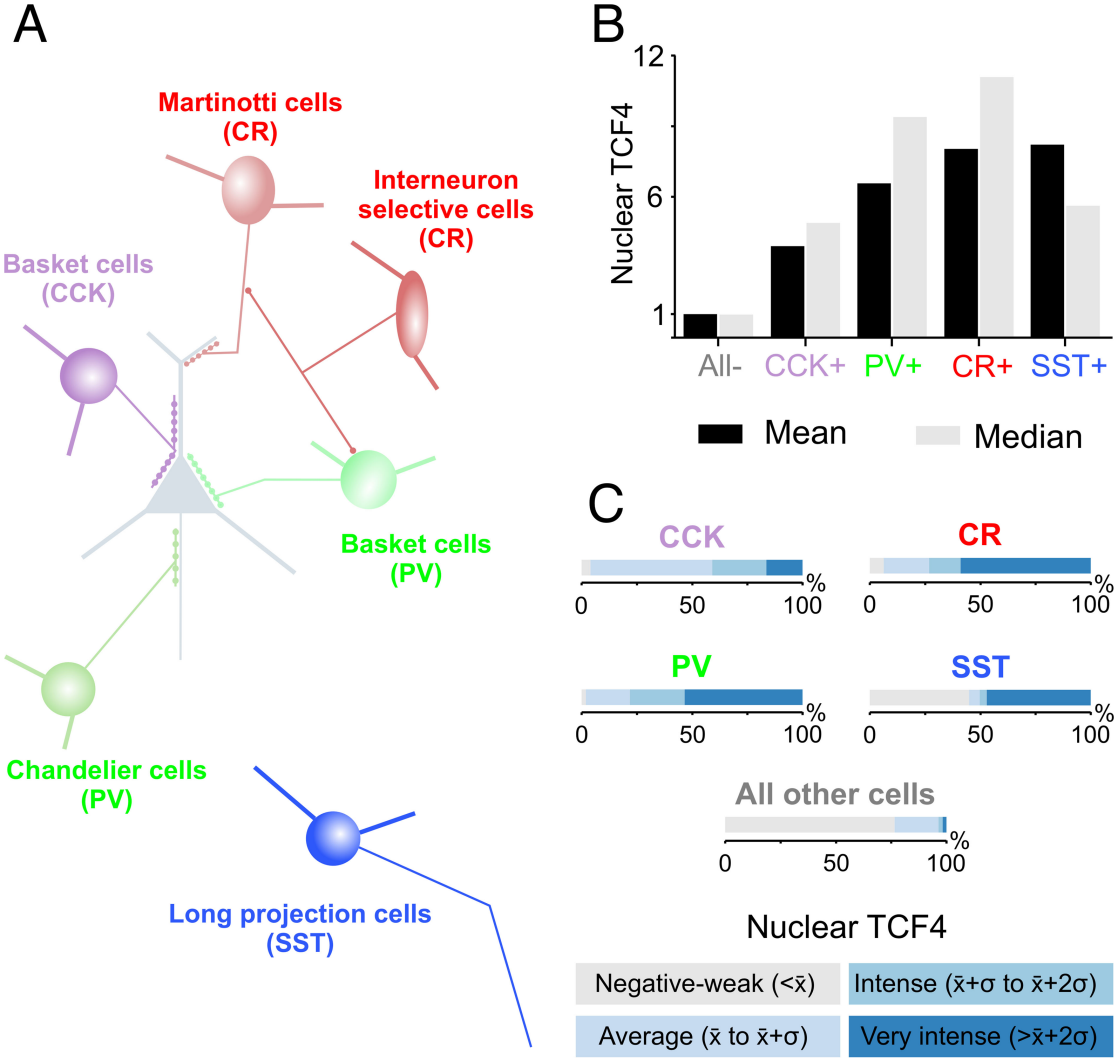
Quantitative analysis of TCF4 expression across GABAergic interneuron subtypes. **(A)** Schematic representation of major interneuron classes and their connectivity patterns: CCK-positive basket cells (pro-cholecystokinin, purple), PV-positive basket and chandelier cells (parvalbumin, green), CR-positive Martinotti and interneuron-selective cells (calretinin, red), and SST-positive long projection cells (somatostatin, blue). **(B)** Mean (black bars) and median (gray bars) nuclear TCF4 intensity for each interneuron subtype compared to marker-negative cells. SST-positive and CR-positive neurons exhibit the highest TCF4 expression (~8-fold above other non-GABAergic cells), followed by PV-positive neurons (~6-fold), while CCK-positive neurons display the lowest levels (~4 fold). **(C)** Distribution analysis showing the proportion of cells in each TCF4 intensity category across interneuron subtypes. SST-positive neurons show a distinctive bimodal distribution with cells clustering in either negative-to-weak or very intense categories. CR-positive and PV-positive neurons have approximately 50% of cells with very intense TCF4 expression, with the remainder more evenly distributed across weak, average, and intense categories. Only a small proportion of proCCK-positive cells show high-intensity TCF4 expression. Analysis based on sextuple immunofluorescence from the temporal cortex of a 5-year-old macaque. Values represent normalized nuclear TCF4 intensity relative to the overall cell population (e.g., all detected DAPI nuclei). Intensity categories were defined relative to the mean (*x̄*) and standard deviation (*σ*) of nuclear TCF4 across all detected nuclei in a given section: negative-to-weak < x̄; average *x̄* to *x̄* +*σ*; intense *x̄* +*σ* to *x̄* +2*σ*; very intense > x̄ +2*σ*.

Our next step was to examine TCF4 expression within subcategories of GABAergic cells. We performed sextuple immunofluorescence labeling for the simultaneous visualization of TCF4 and the nuclear stain DAPI, along with established markers for key GABAergic cell types: parvalbumin (PV), somatostatin (SST), calretinin (CR), and pro-cholecystokinin (proCCK) (Figure 10). This combination of markers provided broad coverage, allowing us to assess TCF4 expression within a majority of GABAergic neurons and across their principal classifications. Quantitative analysis in the 5-year-old neocortex shows that GABAergic neurons express higher TCF4 levels than other cell types (Figure 11). SST-positive and CR-positive neurons exhibit the highest TCF4 levels, approximately 8-fold higher than cells negative for all markers. PV-positive neurons show moderate TCF4 enrichment (6.6-fold), while proCCK-positive neurons have the lowest expression among marker-positive populations, yet still 3.9-fold above baseline.

The distribution analysis revealed additional differences. Most cells for each GABAergic cell type, except proCCK-positive neurons, have an average (x̄ to x̄+*σ*) or intense (x̄+σ to x̄+2σ) TCF4 labeling. CR-positive and PV-positive neurons show similar distributions, with both crossing into intense TCF4 labeling around the 40-45th percentile. SST-positive neurons show a unique distribution pattern with a sharp transition around the 50th percentile: with approximately 45% showing negative-to-weak TCF4 labeling, yet with approximately 47% displaying very intense TCF4 labeling (more than two standard deviations above average, > x̄+2σ).

### 3.4 Public transcriptomic datasets corroborate TCF4 developmental dynamics across the primate brain

To provide broader context for the TCF4 expression patterns observed through *in situ* hybridization and immunofluorescence, we examined developmental dynamics and cell-type specificity using complementary publicly available bulk-tissue and single-nucleus transcriptomic datasets.

**Figure 12:**
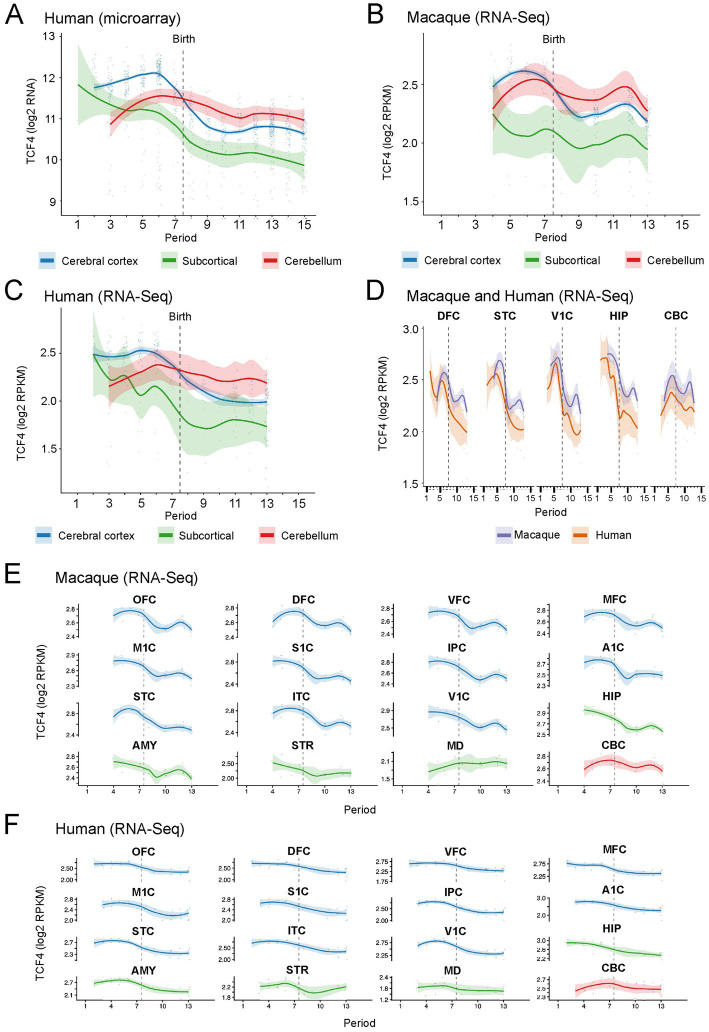
TCF4 mRNA peaks prenatally and declines postnatally across 16 brain regions in human and macaque. Re-analysis of two publicly available developmental brain transcriptomic atlases (Kang et al., 2011, human Affymetrix Human Exon 1.0 ST microarray; Zhu et al., 2018, bulk RNA-seq of human and rhesus macaque brains). **(A)** Developmental trajectory of TCF4 mRNA in the human brain (microarray, Kang et al., 2011). Y-axis: log2 robust multi-array average normalized expression. Each dot represents one tissue sample; solid lines and shaded ribbons are locally estimated scatterplot smoothing (loess) curves (spa*n =* 0.5) and 95% confidence intervals (CI) fitted within each anatomical group: cerebral cortex (blue; 11 neocortical areas: orbital, dorsolateral, ventrolateral, and medial prefrontal cortex; primary motor cortex; primary somatosensory cortex; posterior inferior parietal cortex; primary auditory cortex; superior temporal cortex; inferolateral temporal cortex; primary visual cortex), subcortical (red; hippocampus, amygdala, striatum, mediodorsal nucleus of the thalamus), and cerebellum (green; cerebellar cortex). Light grey shading covers the prenatal periods (P1–P7); the vertical dashed line marks birth (boundary between P7 and P8). **(B)** Developmental trajectory of TCF4 mRNA in rhesus macaque brain (RNA-seq, Zhu et al., 2018). Y-axis: log2 reads per kilobase per million mapped reads (RPKM). Same plotting conventions as in **(A)**. **(C)** Developmental trajectory of TCF4 mRNA in human brain (RNA-seq, Zhu et al., 2018). **(D)** Direct cross-species comparison of TCF4 trajectories between human (orange) and macaque (purple) within the Zhu et al. (2018) dataset, in five matched brain regions: dorsolateral prefrontal cortex, superior temporal cortex, primary visual cortex, hippocampus, and cerebellar cortex. Both species show a prenatal peak and postnatal decline. **(E)** Per-region TCF4 trajectories in macaque RNA-seq (Zhu et al., 2018) across all 16 brain regions. **(F)** Per-region TCF4 trajectories in human (RNA-seq, Zhu et al., 2018) across all 16 brain regions. For human samples, developmental ages were mapped onto the 15 developmental periods defined by Kang et al. (2011): P1, embryonic (4–8 postconceptional weeks [pcw]); P2, early fetal (8–10 pcw); P3, early fetal (10–13 pcw); P4, early mid-fetal (13–16 pcw); P5, early mid-fetal (16–19 pcw); P6, late mid-fetal (19–24 pcw); P7, late fetal (24–38 pcw); P8, neonatal (birth–6 months); P9, early infancy (6–12 months); P10, late infancy (1–3 years); P11, early childhood (3–6 years); P12, middle–late childhood (6–12 years); P13, adolescence (12–20 years); P14, young adulthood (20–40 years); P15, middle adulthood (40–60 years). For the Zhu et al. (2018) dataset, macaque developmental ages were harmonized onto the Kang human period scale using the authors’ pre-computed predicted period assignments, which are based on cross-species developmental modeling rather than direct chronological age matching. In our 366-sample macaque cohort, samples spanned 9 of the 15 Kang periods (P4–P10, P12, P13), with no coverage of the earliest embryonic periods (P1–P3), early childhood (P11), or young-to-middle adulthood (P14, P15). OFC, orbital prefrontal cortex; DFC, dorsolateral prefrontal cortex; VFC, ventrolateral prefrontal cortex; MFC, medial prefrontal cortex; M1C, primary motor cortex; S1C, primary somatosensory cortex; IPC, posterior inferior parietal cortex; A1C, primary auditory cortex; STC, superior temporal cortex; ITC, inferolateral temporal cortex; V1C, primary visual cortex; HIP, hippocampus; AMY, amygdala; STR, striatum; MD, mediodorsal nucleus of the thalamus; CBC, cerebellar cortex.

In the human brain (Kang microarray; 1,281 samples from 56 donors across 16 brain regions), TCF4 mRNA levels peaked during mid-to-late fetal periods (P5-P6, approximately 13–24 postconceptional weeks) in both the cerebral cortex and subcortical structures. They then declined by about twofold through neonatal and early infancy periods (P8-P10) to a stable postnatal plateau maintained into adulthood (Figure 12A). The cerebellar cortex followed a distinct pattern: TCF4 expression did not decline sharply after birth. Instead, it peaked briefly between developmental stages P5 and P7 before stabilizing at levels higher than those in the cortex into adulthood. The same prenatal-peak/postnatal-decline pattern was independently observed in both human and macaque samples from the Zhu et al. (2018) RNA-seq dataset (Figures 12B,C). The human trajectories from the Kang microarray (Figure 12A) and the Zhu RNA-seq (Figure 12C), measured on two independent cohorts and different platforms, showed strong agreement, confirming the robustness of the observed developmental pattern. Direct comparison of human and macaque TCF4 trajectories within the Zhu et al. (2018) dataset showed strong cross-species conservation across matched brain regions (Figure 12D). Both species showed the prenatal peak and a postnatal decline, with the macaque trajectory exhibiting the expected faster developmental timing compared to humans. Per-region trajectory analysis confirmed that the developmental decline was consistent across neocortical, subcortical, and cerebellar regions in both species (Figures 12E,F). One exception was the mediodorsal thalamus, where macaque TCF4 expression seemed to increase after birth, unlike in humans, where it decreased. However, this apparent species difference should be interpreted with caution. Prenatal macaque sampling for MD was sparse, with only 5 samples distributed across periods 4–6, and one of these was a clear low-expression outlier (RPKM 1.5 versus 3.3 for the other sample at the same period), which likely contributes to the apparent prenatal-to-postnatal increase. Whether this reflects a genuine interspecies difference in thalamic TCF4 regulation or a sampling artifact remains unclear. Overall, these bulk transcriptomic data provide independent, multi-region, cross-species support for the developmental dynamics of TCF4 demonstrated by our immunohistochemical analysis.

### 3.5 Single-nucleus RNA-seq confirms brain-wide GABAergic enrichment and replicates the interneuron-subtype hierarchy

**Figure 13:**
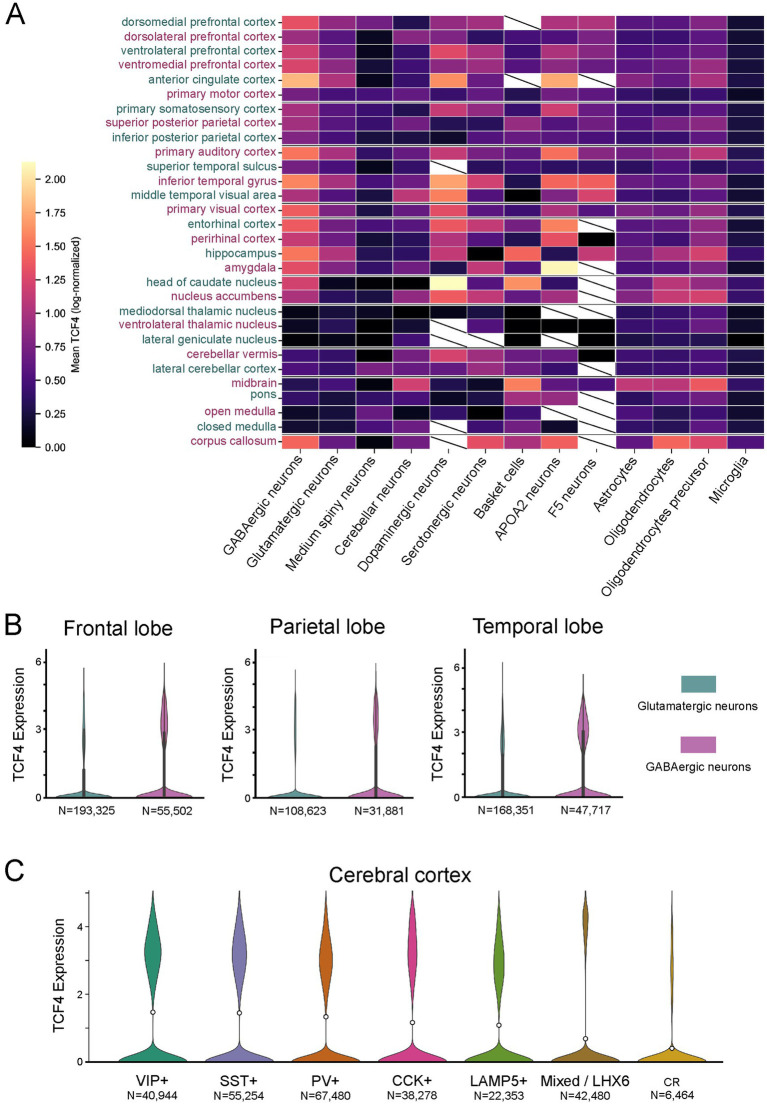
TCF4 is enriched in GABAergic neurons brain-wide. Extended analysis of the Chiou et al. (2023) adult rhesus macaque snRNA-seq atlas (single-cell combinatorial-indexing RNA-seq [sci-RNA-seq3]; four 10-year-old male donors), using a brain-wide 1.5 million-cell subset (30 brain regions, 17 cell classes) and a dedicated 371,548-cell GABAergic-neuron subset. **(A)** Heatmap of mean TCF4 expression (log-normalized counts) across 30 brain regions (rows) and cell classes (columns). GABAergic neurons show the highest mean TCF4 of the 17 cell classes (mean 0.99), while microglia (mean 0.30) and medium spiny neurons (mean 0.13) show the lowest neuronal expression. **(B)** TCF4 expression in GABAergic versus glutamatergic neurons across 3 cortical regions (frontal, parietal, temporal) shows higher TCF4 in GABAergic populations. Cell counts are shown beneath each violin. **(C)** TCF4 expression by interneuron subtype in macaque neocortex (*n =* 273,253 cells from four cortical regions). Subtypes were identified *de novo* by marker-gene signature analysis of the 20 deposited GABAergic subclusters. Subtypes are ordered by descending mean TCF4: vasoactive intestinal peptide (VIP)-positive (*n =* 40,944; mean 1.47), somatostatin (SST)-positive (*n =* 55,254; mean 1.45), parvalbumin (PV)-positive (*n =* 67,480; mean 1.33), cholecystokinin (CCK)-positive (*n =* 38,278; mean 1.17), lysosomal-associated membrane protein 5 (LAMP5)-positive (*n =* 22,353; mean 1.09), mixed/LIM homeobox 6 (LHX6) (*n =* 42,480; mean 0.68), and calbindin 2 (i.e., calretinin; CR)-positive (*n =* 6,464; mean 0.40).

To evaluate whether the GABAergic enrichment of TCF4 extends beyond the temporal cortex examined by immunofluorescence, we analyzed publicly available snRNA-seq data from the adult rhesus macaque brain (Chiou et al., 2023). We first looked at TCF4 expression across all 30 sampled brain regions and all 17 annotated cell classes using the 1.5 million-cell subset from the Chiou et al. atlas (Figure 13A). Among the 17 cell classes, GABAergic neurons showed the highest overall mean TCF4 expression across the brain (mean log-normalized TCF4 = 0.99; 31.2% of cells TCF4-positive), followed by ependymal cells (0.97), oligodendrocyte precursor cells (0.87), and oligodendrocytes (0.79). Glutamatergic neurons ranked seventh (mean 0.72), and microglia showed low expression (mean 0.30; 8.4% of cells positive), consistent with the absence of detectable TCF4 immunoreactivity in TMEM119-positive microglia. Interestingly, medium spiny neurons, which are GABAergic projection neurons in the striatum, showed the lowest TCF4 levels of any neuronal class (mean 0.13; 4.6% positive), suggesting that the GABAergic enrichment of TCF4 is specific to telencephalic interneurons rather than a feature of all GABAergic populations.

Focusing the analysis on neurons labeled as GABAergic or glutamatergic within the three cortical regions showed consistently higher TCF4 expression in GABAergic populations across all examined regions aligning with the cell-type-specific pattern observed in our primary immunohistochemical analysis (Figure 13B).

To test whether the interneuron-subtype TCF4 hierarchy observed by immunofluorescence is also seen at the transcriptomic level, we analyzed 273,253 neocortical GABAergic nuclei from the Chiou et al. (2023) GABAergic subset. Interneuron subtypes were identified *de novo* using marker-gene signature analysis. The TCF4 ranking across subtypes showed VIP-positive (mean 1.47) and SST-positive (1.45) as the highest, followed by PV-positive (1.33), CCK-positive (1.17), LAMP5-positive (1.09), Mixed/LHX6 (0.68), and CR-positive (0.40) (Figure 13C). The ranking SST > PV > CCK matches the quantitative immunofluorescence data in Figure 11, independently confirmed across all neocortical regions—not just temporal cortex—in a separate cohort of four macaque donors using a different method. VIP-positive interneurons, which were not directly assessed by immunohistochemistry in this study, ranked highest in the transcriptomic ranking, consistent with rodent transcriptomic data showing TCF4 enrichment in the VIP-positive cluster (Tasic et al., 2016). The CR-positive subtype showed lower mean TCF4 than the CR-positive population in Figure 11; this likely reflects the fact that anti-calretinin immunohistochemistry labels both VIP-positive/CR-positive interneuron-specific cells (which have high TCF4 and are assigned to the VIP-positive transcriptomic subtype) and VIP-negative/CR-positive populations, whereas transcriptomic subclustering separates these two populations.

## 4 Discussion

In this study, we provide a detailed analysis of TCF4 expression across cell types in the postnatal nonhuman primate neocortex. Our observations show that TCF4 is predominantly neuronal in the postnatal macaque neocortex, with little to no expression in microglia, oligodendrocytes, and most astrocytes. In late gestation, TCF4 is broadly expressed in neurons. As the neocortex matures, however, expression shifts toward GABAergic neurons, such that by young adulthood, TCF4 is most enriched in specific GABAergic interneuron subtypes. These findings provide a neuroanatomical framework for understanding TCF4’s role in cortical development and its contributions to disorders.

### 4.1 TCF4 expression is predominantly neuronal and low in glia during postnatal life

We observed consistently low levels of TCF4 in glial populations, including astrocytes, oligodendrocytes, and microglia. Microglia lacked detectable TCF4, consistent with findings from rodent TCF4-GFP reporter studies (Kim et al., 2020). Mature oligodendrocytes and astrocytes displayed only weak expression, suggesting that although TCF4 is required for oligodendrocyte and astrocyte precursor differentiation (Phan et al., 2020; Wedel et al., 2020; Zhang et al., 2021; Zhang et al., 2024), its importance diminishes as these cells mature. The pronounced myelin abnormalities observed in PTHS (Phan et al., 2020) likely stem from oligodendrocyte vulnerability during prenatal stages, when TCF4 expression is higher. A small subset of SOX9-positive astrocytes showed TCF4 levels comparable to GABAergic neurons, raising the possibility of specialized astrocyte populations with TCF4-dependent functions. In contrast, neurons showed robust, but heterogeneous expression, suggesting that TCF4 plays a predominantly neuronal role in the postnatal primate neocortex, while its glial functions are most critical during development.

### 4.2 Developmental transition to GABAergic neuron enrichment

TCF4 expression in the primate temporal cortex undergoes a striking reorganization across development. During late gestation, expression is broad, spanning nearly all neuronal lineages. Postnatally, this widespread pattern shifts: TCF4 gradually declines in excitatory neurons while remaining high and becomes selectively enriched in GABAergic interneurons. By five years of age, the “very intense” (>x̄+2σ) TCF4-positive population is almost exclusively inhibitory. This enrichment varies among subtypes. Using immunofluorescence, SST-positive and CR-positive neurons showed the highest nuclear TCF4 intensities, while PV-positive neurons showed moderate levels, and proCCK-positive neurons had the lowest. It is worth noting that anti-CR immunohistochemistry labels a heterogeneous population that includes both VIP-positive/CR-positive interneuron-selective cells, which have high TCF4 levels, and VIP-negative/CR-positive populations; transcriptomic clustering separates these two groups. The TCF4 enrichment observed in CR-positive cells by immunohistochemistry, therefore, likely reflects predominant labeling of the VIP-positive/interneuron-selective subset.

The hierarchy raises the possibility that TCF4 may have different regulatory roles across inhibitory lineages, possibly related to the varying physiological needs of somatodendritic- versus perisomatic-targeting cells. Overall, this developmental pathway reflects the extended maturation process of primate inhibitory circuits (Hashimoto et al., 2009; Hoftman and Lewis, 2011; Chung et al., 2017; Hosseini Fin et al., 2025).

The broad gestational expression likely reflects TCF4’s involvement in early neurodevelopmental processes — progenitor proliferation, neuronal differentiation, and laminar positioning (Chen et al., 2016; Li et al., 2019). In excitatory neurons, TCF4 regulates early maturation programs, including voltage-gated channel scaling and dendritic spine morphology (Rannals et al., 2016; Crux et al., 2018). As these neurons complete structural maturation, ongoing TCF4 activity appears dispensable, consistent with the postnatal decline we observed in NeuN-positive/GAD-negative cells. The postnatal interneuron enrichment may instead reflect roles in critical-period processes such as dendritic arborization and synaptic integration, potentially through TCF4’s function as a heterodimeric partner of ASCL1 and NeuroD family members (Fischer et al., 2014), and its regulation of downstream targets including voltage-gated sodium channel subunits (Rannals et al., 2016).

### 4.3 Transcriptomic support for TCF4 cellular distribution

The transcriptomic re-analyses agree with the immunohistochemical and *in situ* hybridization results on all key points while broadening their scope. The prenatal peak and postnatal decrease of TCF4 protein, as shown by immunofluorescence in macaque temporal cortex, are reflected at the mRNA level across 16 brain regions and both species in the Kang et al. (2011) and Zhu et al. (2018) bulk atlases, confirming that this pattern is a conserved, widespread feature of primate brain development rather than a property of a single cortical area examined histologically. Postnatal GABAergic enrichment of TCF4 is also evident in snRNA-seq data, where GABAergic neurons express the highest average TCF4 levels across all 30 sampled regions. The interneuron-subtype hierarchy identified by immunofluorescence (SST > PV > CCK) is mirrored at the transcriptomic level (Figure 13C). As previously noted, CR-positive interneurons detected via immunohistochemistry represent a mixed population that transcriptomic clustering separates into VIP-positive (high TCF4) and CR-positive (lower TCF4) subtypes, explaining the apparent discrepancy in their rankings. Importantly, VIP-positive interneurons, not directly evaluated by immunohistochemistry in this study, ranked highest in the snRNA-seq analysis (mean log-normalized TCF4 = 1.47), which is consistent with rodent transcriptomic data (Tasic et al., 2016). The low TCF4 signal in microglia seen by immunohistochemistry is likewise confirmed, with microglia displaying the lowest average TCF4 among major cell classes in the snRNA-seq atlas.

Interestingly, the medium spiny neuron population of the striatum, which, although GABAergic, has the lowest TCF4 levels among all neuronal classes in the snRNA-seq atlas (mean 0.13; 4.6% positive). This dissociation between neurotransmitter identity and TCF4 enrichment suggests that TCF4’s high expression in cortical interneurons may reflect their shared developmental origin from the ganglionic eminence rather than GABAergic neurotransmission per se.

For the cerebellum, different techniques produce less consistent results. Both bulk atlases show persistent TCF4 mRNA into adulthood, matching our previous immunohistochemical evidence of strong TCF4 protein in granule cells (Burette et al., 2024). However, the Chiou et al. (2023) snRNA-seq data show unexpectedly low cerebellar expression. This likely reflects the low per-cell transcript capture of the sci-RNA-seq3 platform, which systematically underestimates transcript counts in small-nuclei populations such as cerebellar granule cells (Cao et al., 2019; Ding et al., 2020), rather than genuinely low cerebellar TCF4.

### 4.4 Evolutionary considerations

By characterizing TCF4 expression in macaque neocortex, our study provides insight into the conservation and potential divergence of TCF4 function across species. In adult mouse cortex, TCF4 is enriched in GABAergic neurons, particularly parvalbumin-positive interneurons (Tasic et al., 2016; Ling et al., 2020), consistent with epigenomic data showing enrichment of TCF4 binding motifs within ATAC-seq peaks of parvalbumin neurons. In PTHS mouse models, Tcf4 mutations reduce the density of PV- and VIP-positive cells (Chen et al., 2023). Our primate data align with these rodent findings, suggesting evolutionary conservation of TCF4’s cell-type-specific roles within the inhibitory network, with VIP- and SST-positive interneurons consistently showing the highest TCF4 burden across both rodents and primates.

### 4.5 Implications for Pitt-Hopkins syndrome and psychiatric diseases

The developmental shift of TCF4 expression toward inhibitory neurons provides a cellular framework for understanding disease mechanisms. Many symptoms of Pitt-Hopkins syndrome likely originate from TCF4’s broad developmental roles during prenatal brain formation. Still, our findings suggest that its enrichment in GABAergic neurons may also contribute to postnatal phenotypes such as seizures, consistent with the established importance of inhibitory circuits in seizure regulation (Magloire et al., 2019). The preferential expression of TCF4 in mature interneurons is also relevant to psychiatric disorders linked to *TCF4* variants, where cortical inhibitory dysfunction is a common pathological feature. In major depressive disorder, for example, reduced GABAergic signaling correlates with symptom severity, while normalization associates with remission (Cutler et al., 2023). Altered parvalbumin interneuron activity has been implicated in post-traumatic stress disorder (Huang et al., 2023; Su et al., 2025), and both schizophrenia and bipolar disorder are characterized by interneuron pathology, particularly affecting PV- and SST-positive populations (Taylor and Tso, 2015; Gigase et al., 2019; Krolewski et al., 2025; Morris et al., 2008; Jiang et al., 2013; Van Derveer et al., 2021; Dienel et al., 2023; Marin, 2024) Thus, the heightened expression of TCF4 in these interneuron subtypes aligns with known vulnerability points in psychiatric disease. Even subtle alterations in TCF4 dosage or function within mature interneurons may destabilize cortical excitatory–inhibitory balance, impair synaptic plasticity, and compromise cognition, thereby increasing susceptibility to neurodevelopmental and psychiatric conditions.

These observations have direct implications for Pitt-Hopkins syndrome therapeutics. Because mature interneurons — particularly VIP-, SST-, and PV-positive subtypes — represent the principal sites of TCF4 activity in the adult primate cortex, they constitute compelling cellular targets for gene reinstatement approaches. Precise dosage control will be critical, as both over- and under-expression of TCF4 can be detrimental to inhibitory circuits (Brzózka et al., 2010; Badowska et al., 2020). Strategies that harness endogenous *TCF4* promoters and enhancers, or emerging gene-enhancement tools such as CRISPRa, may provide more physiologically regulated control than constitutive overexpression approaches. Recent demonstrations of successful CRISPR-based *TCF4* activation (Papes et al., 2022) underscore the feasibility of this direction.

### 4.6 Methodological considerations and limitations

While our findings provide a detailed cellular map of TCF4 expression in the macaque neocortex, several limitations should be acknowledged. Immunofluorescence intensity may not perfectly reflect protein abundance, particularly across different developmental stages where tissue quality and antigen accessibility vary. Furthermore, although the antibody’s antigen target theoretically enables recognition of all TCF4 isoforms, differential binding affinities across isoforms are possible. Such variability could introduce systematic bias in observed staining patterns, potentially skewing our interpretation of cell-type-specific or developmental-stage-specific expression. The limited number of animals at each developmental stage reflects inherent constraints in nonhuman primate research and prevents fully-powered statistical comparisons across ages and cell subtypes. To facilitate descriptive analysis, we used categorical intensity classifications (“weak, average, intense, very intense”) as standardized descriptive tools, with thresholds defined relative to each section’s mean and variance. While these categories mirror qualitative impressions of staining intensity, they are descriptive rather than inferential and are not substitutes for statistical inference.

Future work with emerging spatial transcriptomic platforms like MERFISH, Xenium, or DART-FISH applied to macaque neocortex across development could enable the simultaneous quantification of TCF4 alongside dozens of cell-type markers at single-cell resolution, providing a more complete picture of the trajectory described here.

## 5 Conclusion

In summary, we show that TCF4 expression undergoes a developmental transition from broad neuronal distribution in early stages to selective enrichment in inhibitory interneurons during primate cortical maturation. Within interneurons, SST and CR populations exhibit the highest TCF4 expression, positioning these cells as key mediators of TCF4 function in the mature cortex. These results provide a neuroanatomical framework for understanding how *TCF4* haploinsufficiency contributes to Pitt-Hopkins syndrome and how common variants increase psychiatric disease risk. By identifying interneuron subtypes as principal sites of TCF4 activity, this work highlights new avenues for targeted investigation of TCF4-related disease mechanisms.

## Data Availability

The original contributions presented in the study are included in the article/supplementary material, further inquiries can be directed to the corresponding author.

## Footnotes

1
http://evolution.psychencode.org

## Glossary

A1C: Primary auditory cortex
AMY: Amygdala
bHLH: Basic helix–loop–helix
CBC: Cerebellar cortex
CCK: Cholecystokinin
CGE: Caudal ganglionic eminence
CNS: Central nervous system
CR: Calretinin
DFC: Dorsolateral frontal cortex
GABA: Gamma-aminobutyric acid
GAD: Glutamic acid decarboxylase
GD: Gestation day
GFAP: Glial fibrillary acidic protein
HCR-FISH: Hybridization chain reaction fluorescent *in situ* hybridization
HIP: Hippocampus
IPC: Inferior posterior parietal cortex
ITC: Inferolateral temporal cortex
ITF2: Immunoglobulin transcription factor 2
LAMP5: Lysosomal-associated membrane protein 5
LHX6: LIM homeobox 6
M1C: Primary motor cortex
MD: Mediodorsal nucleus of the thalamus
MERFISH: Multiplexed error-robust fluorescence in situ hybridization
MFC: Medial frontal cortex
MGE: Medial ganglionic eminence
NeuN: Neuronal nuclear protein
NHP: Nonhuman primate
OFC: Orbitofrontal cortex
OLIG2: Oligodendrocyte transcription factor 2
PB: Phosphate buffer
PBS: Phosphate-buffered saline
PBS-T: Phosphate-buffered saline with Triton X-100
pcw: Postconceptional weeks
PTHS: Pitt-Hopkins syndrome
PTSD: Post-traumatic stress disorder
PV: Parvalbumin
RMA: Robust multi-array average
RNA-seq: RNA sequencing
RPKM: Reads per kilobase per million mapped reads
S1C: Primary somatosensory cortex
sci-RNA-seq3: Single-cell combinatorial-indexing RNA sequencing (third generation)
SEF2: Site-specific factor 2
snRNA-seq: Single-nucleus RNA sequencing
SOX9: SRY-box transcription factor 9
SST: Somatostatin
STC: Superior temporal cortex
STR: Striatum
TCF4: Transcription factor 4
TMEM119: Transmembrane protein 119
TSA: Tyramide signal amplification
UBE3A: Ubiquitin protein ligase E3A
V1C: Primary visual cortex
VGAT: Vesicular GABA transporter
VFC: Ventrolateral frontal cortex
VGLUT1: Vesicular glutamate transporter 1
VIP: Vasoactive intestinal peptide
WM: White matter

## Intensity-category definitions:

Negative to weak: Below average, < x̄.

Average: Between the mean and one standard deviation above the mean, x̄ to x̄ + *σ*.

Intense: Between one and two standard deviations above the mean, x̄ + σ to x̄ + 2σ.

Very intense: More than two standard deviations above the mean, > x̄ + 2σ.
